# The Physician Himself

**Published:** 1884-06

**Authors:** 


**The Physician Himself**: What he should add to his scientific acquirements. By D. W. Cathell, M. D., late Professor of Pathology in the College of Physicians and Surgeons of Baltimore, etc. Second edition, carefully revised. Baltimore: Cushings & Bailey, 262 W. Baltimore Street.

We are glad to welcome a second edition of this little work. That a second edition has been so soon called for is satisfactory evidence of its value. We have only to renew the commendation which we expressed in our previous notice of the book. The author has availed himself of the opportunity of a revisal to make some changes and additions which are of value.

